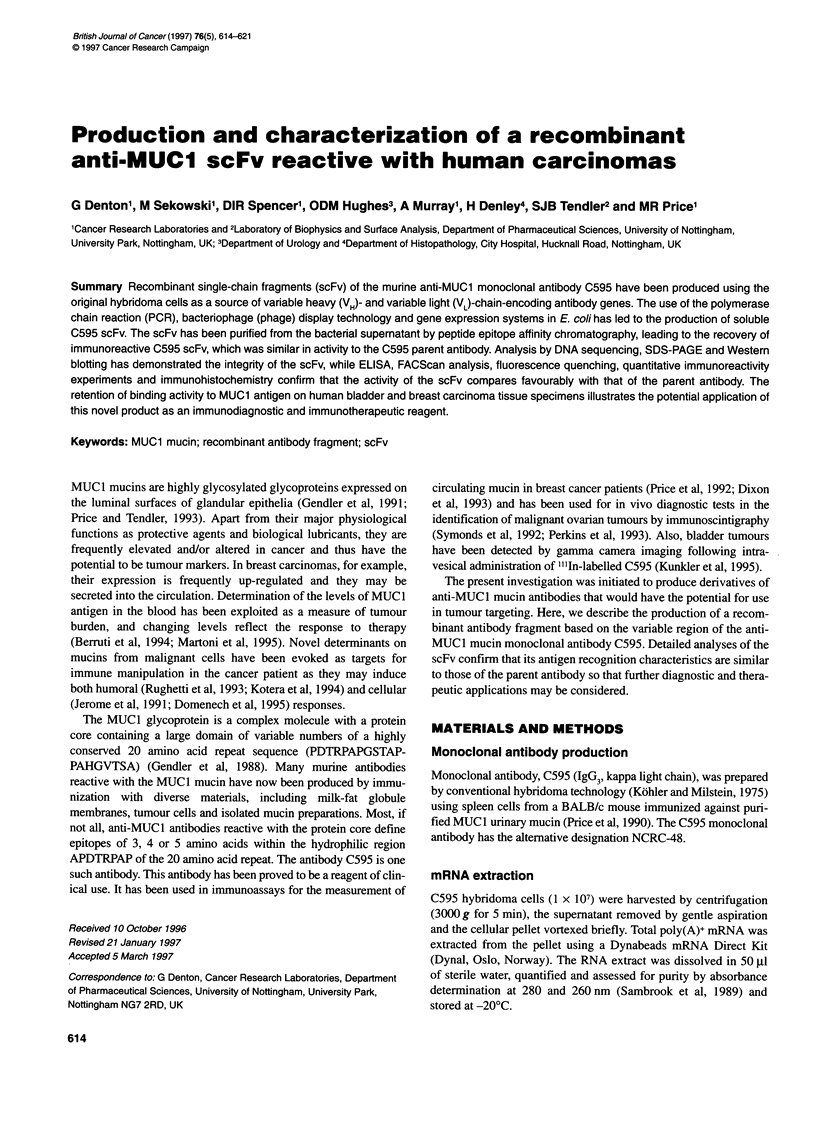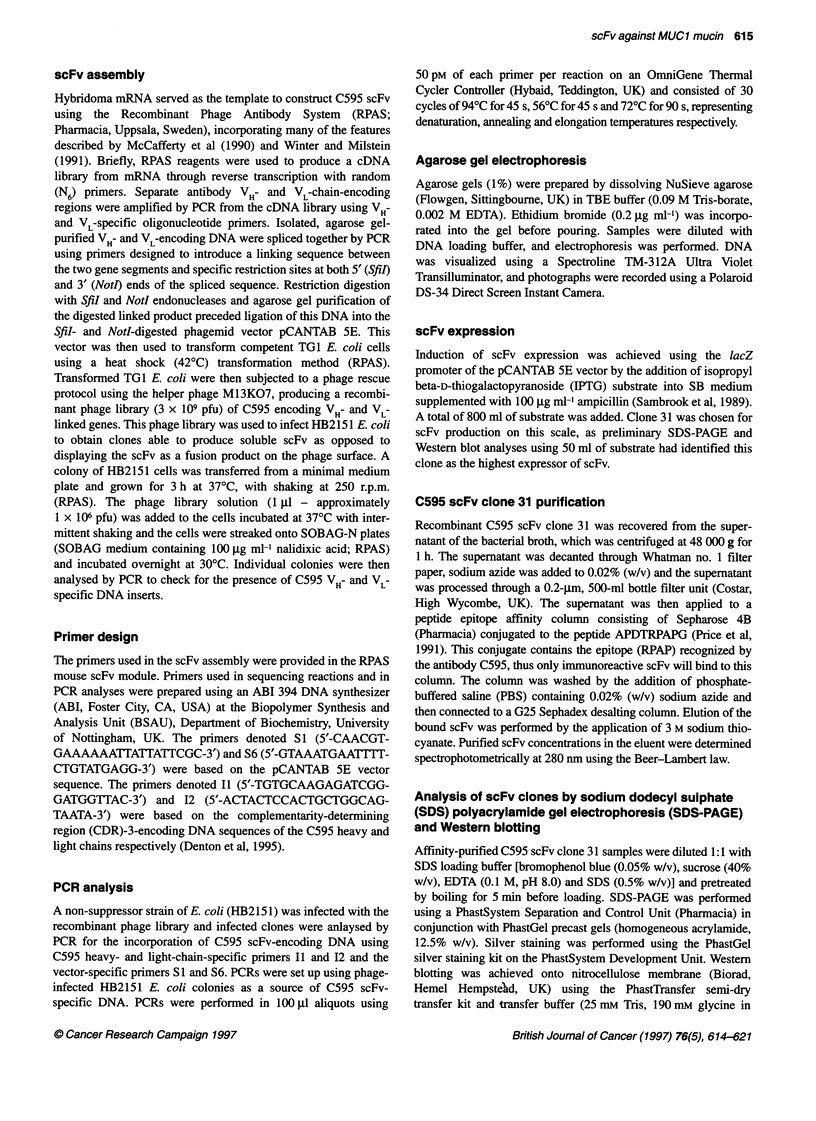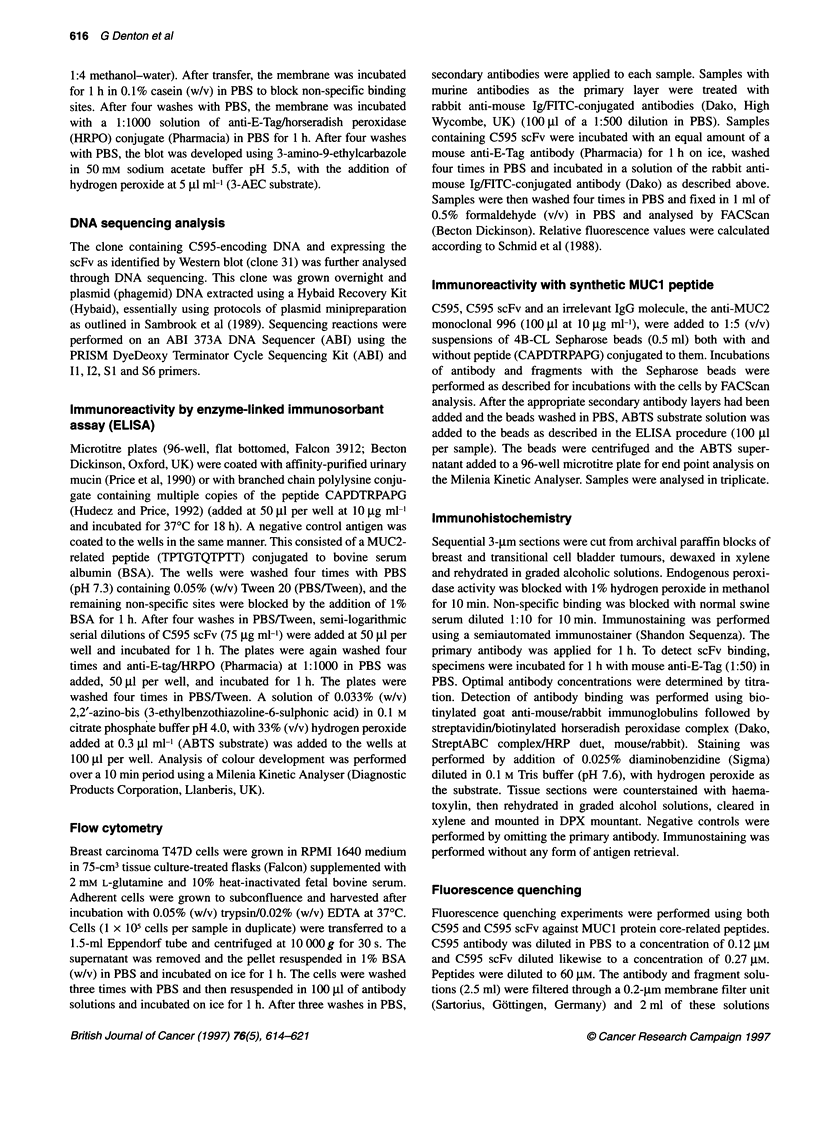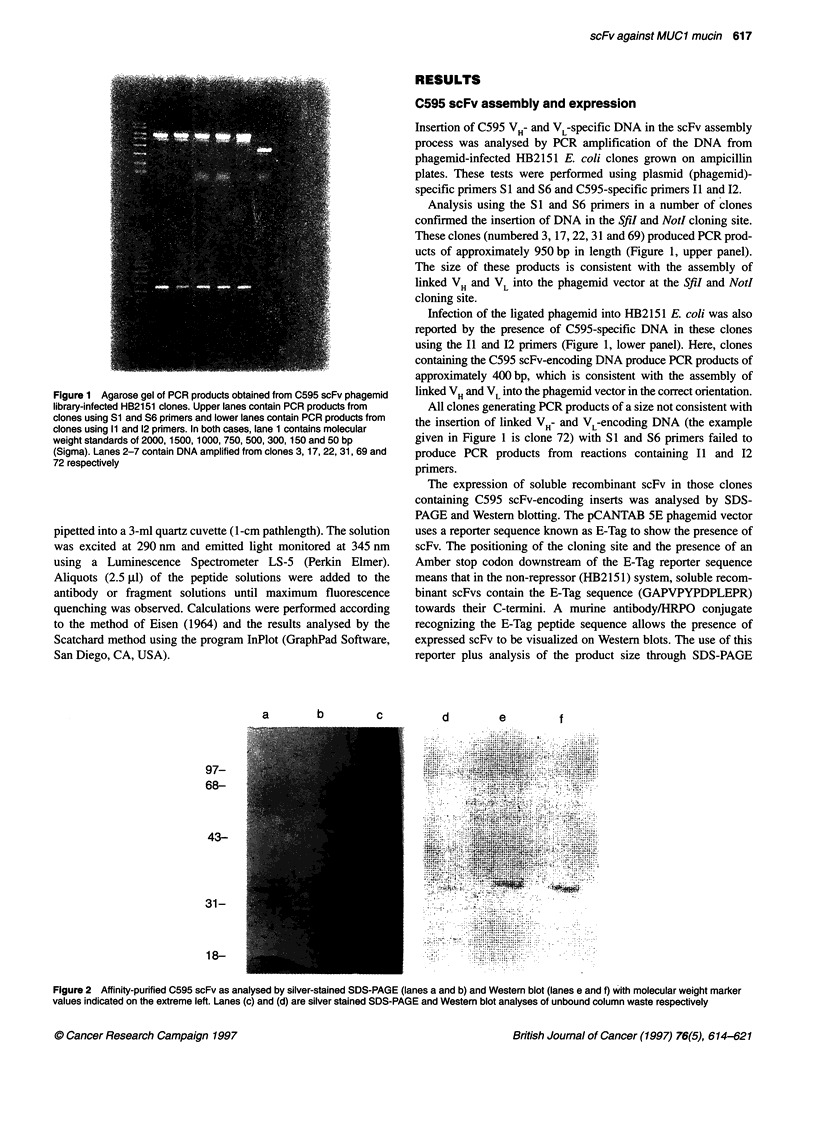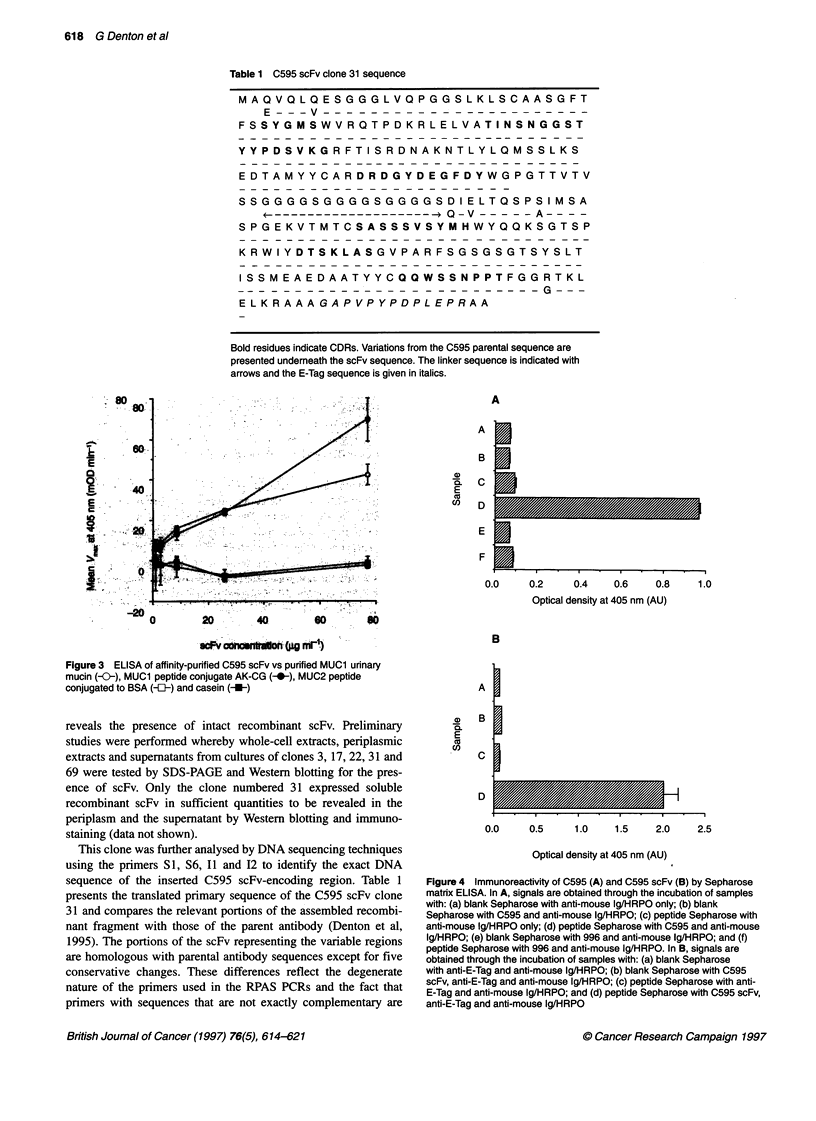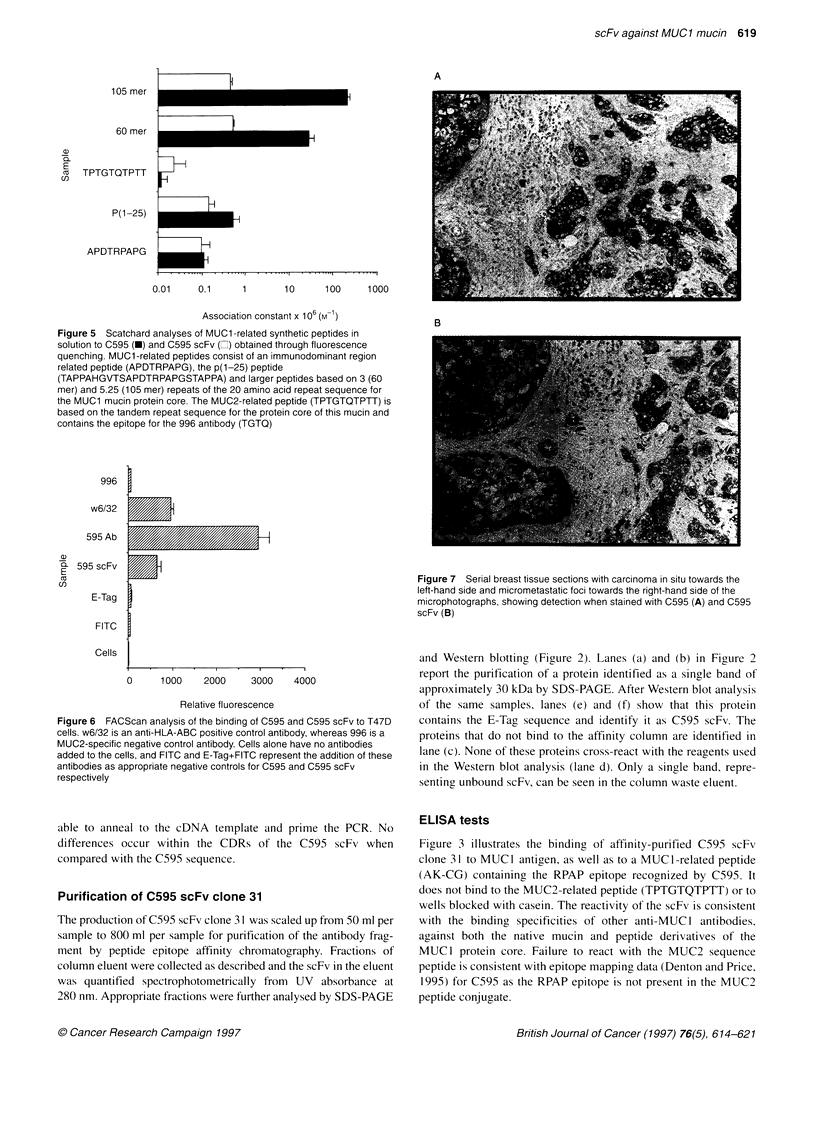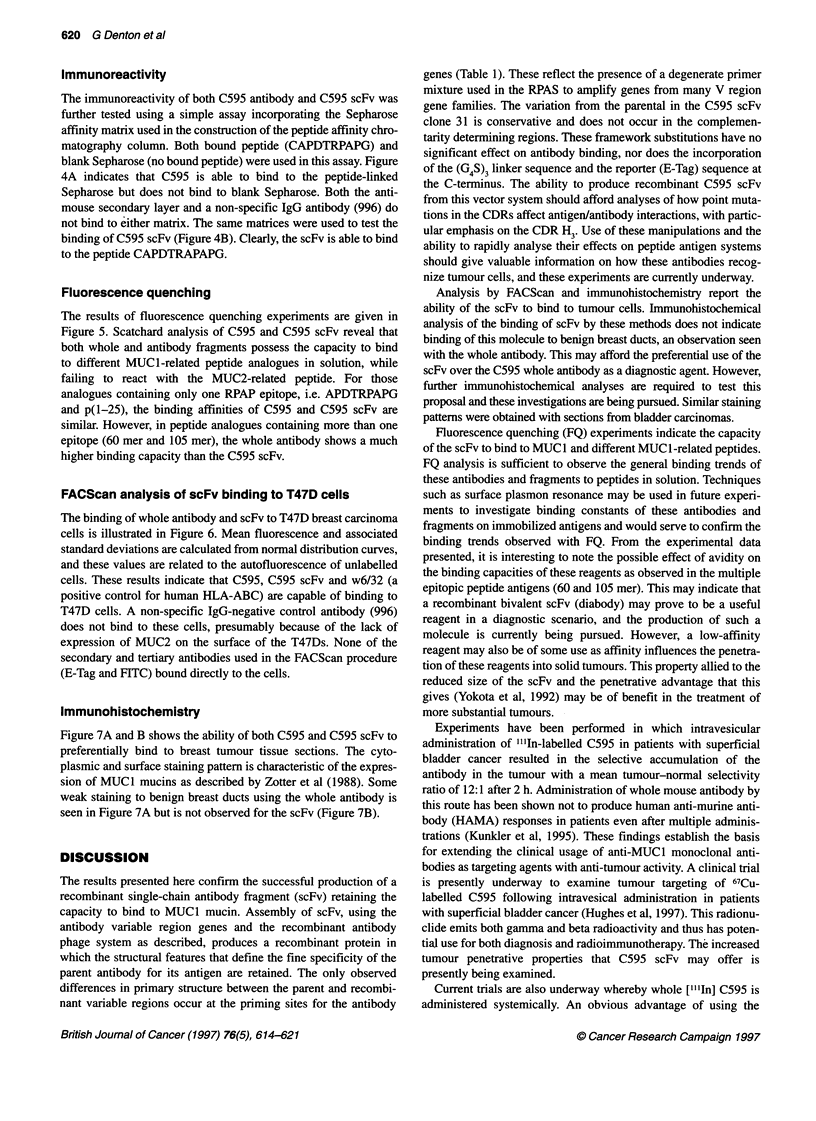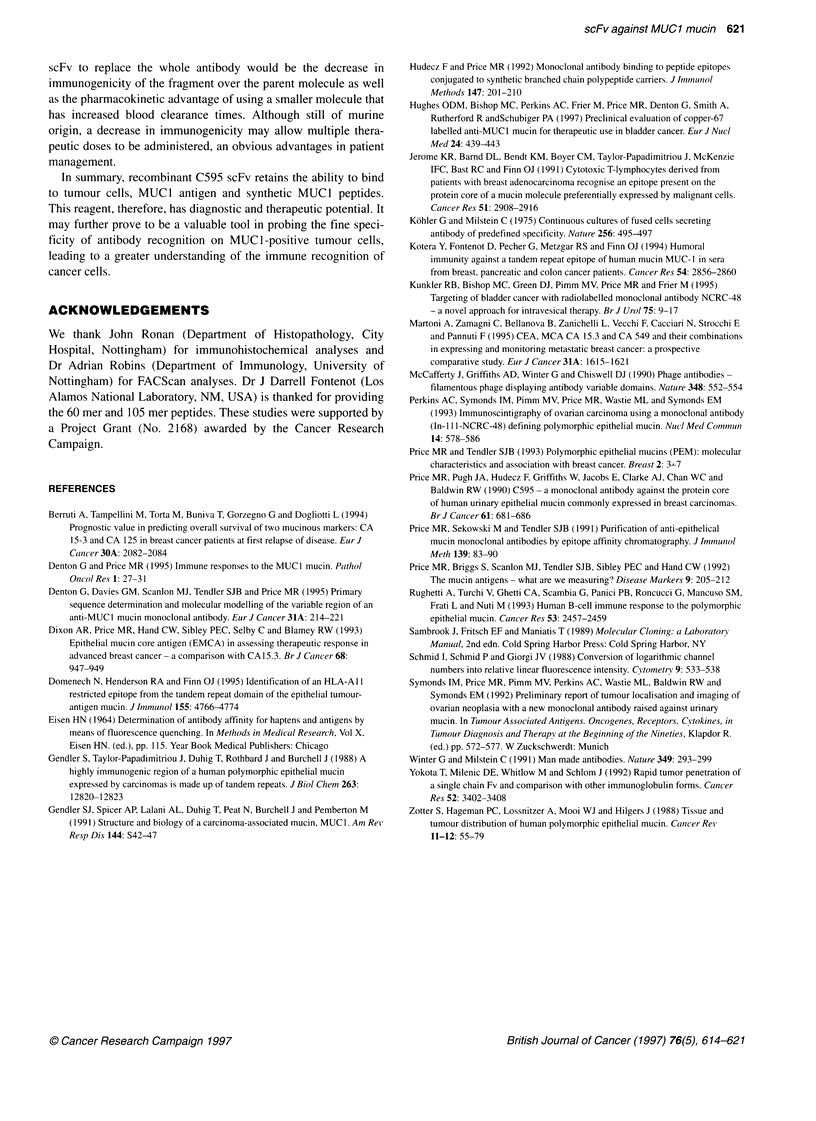# Production and characterization of a recombinant anti-MUC1 scFv reactive with human carcinomas.

**DOI:** 10.1038/bjc.1997.434

**Published:** 1997

**Authors:** G. Denton, M. Sekowski, D. I. Spencer, O. D. Hughes, A. Murray, H. Denley, S. J. Tendler, M. R. Price

**Affiliations:** Cancer Research Laboratories, Department of Pharmaceutical Sciences, University of Nottingham, University Park, UK.

## Abstract

**Images:**


British Journal of Cancer (1997) 76(5), 614–621
© 1997 Cancer Research Campaign

### Production and characterization of a recombinant anti-MUC1 scFv reactive with human carcinomas

**G Denton[1], M Sekowski[1], DIR Spencer[1], ODM Hughes[3], A Murray[1], H Denley[4], SJB Tendler[2] and MR Price[1]**

[1]Cancer Research Laboratories and [2]Laboratory of Biophysics and Surface Analysis, Department of Pharmaceutical Sciences, University of Nottingham, University Park, Nottingham, UK; [3]Department of Urology and [4]Department of Histopathology, City Hospital, Hucknall Road, Nottingham, UK

**Summary** Recombinant single-chain fragments (scFv) of the murine anti-MUC1 monoclonal antibody C595 have been produced using the original hybridoma cells as a source of variable heavy ($V_H$)- and variable light ($V_L$)-chain-encoding antibody genes. The use of the polymerase chain reaction (PCR), bacteriophage (phage) display technology and gene expression systems in *E. coli* has led to the production of soluble C595 scFv. The scFv has been purified from the bacterial supernatant by peptide epitope affinity chromatography, leading to the recovery of immunoreactive C595 scFv, which was similar in activity to the C595 parent antibody. Analysis by DNA sequencing, SDS-PAGE and Western blotting has demonstrated the integrity of the scFv, while ELISA, FACScan analysis, fluorescence quenching, quantitative immunoreactivity experiments and immunohistochemistry confirm that the activity of the scFv compares favourably with that of the parent antibody. The retention of binding activity to MUC1 antigen on human bladder and breast carcinoma tissue specimens illustrates the potential application of this novel product as an immunodiagnostic and immunotherapeutic reagent.

**Keywords:** MUC1 mucin; recombinant antibody fragment; scFv

MUC1 mucins are highly glycosylated glycoproteins expressed on the luminal surfaces of glandular epithelia (Gendler et al, 1991; Price and Tendler, 1993). Apart from their major physiological functions as protective agents and biological lubricants, they are frequently elevated and/or altered in cancer and thus have the potential to be tumour markers. In breast carcinomas, for example, their expression is frequently up-regulated and they may be secreted into the circulation. Determination of the levels of MUC1 antigen in the blood has been exploited as a measure of tumour burden, and changing levels reflect the response to therapy (Berruti et al, 1994; Martoni et al, 1995). Novel determinants on mucins from malignant cells have been evoked as targets for immune manipulation in the cancer patient as they may induce both humoral (Rughetti et al, 1993; Kotera et al, 1994) and cellular (Jerome et al, 1991; Domenech et al, 1995) responses.

The MUC1 glycoprotein is a complex molecule with a protein core containing a large domain of variable numbers of a highly conserved 20 amino acid repeat sequence (PDTRPAPGSTAP-PAHGVTSA) (Gendler et al, 1988). Many murine antibodies reactive with the MUC1 mucin have now been produced by immunization with diverse materials, including milk-fat globule membranes, tumour cells and isolated mucin preparations. Most, if not all, anti-MUC1 antibodies reactive with the protein core define epitopes of 3, 4 or 5 amino acids within the hydrophilic region APDTRPAP of the 20 amino acid repeat. The antibody C595 is one such antibody. This antibody has been proved to be a reagent of clinical use. It has been used in immunoassays for the measurement of circulating mucin in breast cancer patients (Price et al, 1992; Dixon et al, 1993) and has been used for in vivo diagnostic tests in the identification of malignant ovarian tumours by immunoscintigraphy (Symonds et al, 1992; Perkins et al, 1993). Also, bladder tumours have been detected by gamma camera imaging following intravesical administration of $^{111}$In-labelled C595 (Kunkler et al, 1995).

The present investigation was initiated to produce derivatives of anti-MUC1 mucin antibodies that would have the potential for use in tumour targeting. Here, we describe the production of a recombinant antibody fragment based on the variable region of the anti-MUC1 mucin monoclonal antibody C595. Detailed analyses of the scFv confirm that its antigen recognition characteristics are similar to those of the parent antibody so that further diagnostic and therapeutic applications may be considered.

*Received 10 October 1996*
*Revised 21 January 1997*
*Accepted 5 March 1997*

*Correspondence to:* G Denton, Cancer Research Laboratories, Department of Pharmaceutical Sciences, University of Nottingham, University Park, Nottingham NG7 2RD, UK

### MATERIALS AND METHODS

### Monoclonal antibody production

Monoclonal antibody, C595 ($IgG_3$, kappa light chain), was prepared by conventional hybridoma technology (Köhler and Milstein, 1975) using spleen cells from a BALB/c mouse immunized against purified MUC1 urinary mucin (Price et al, 1990). The C595 monoclonal antibody has the alternative designation NCRC-48.

### mRNA extraction

C595 hybridoma cells ($1 \times 10^7$) were harvested by centrifugation (3000 *g* for 5 min), the supernatant removed by gentle aspiration and the cellular pellet vortexed briefly. Total poly(A)$^+$ mRNA was extracted from the pellet using a Dynabeads mRNA Direct Kit (Dynal, Oslo, Norway). The RNA extract was dissolved in 50 μl of sterile water, quantified and assessed for purity by absorbance determination at 280 and 260 nm (Sambrook et al, 1989) and stored at –20°C.

### scFv assembly

Hybridoma mRNA served as the template to construct C595 scFv using the Recombinant Phage Antibody System (RPAS; Pharmacia, Uppsala, Sweden), incorporating many of the features described by McCafferty et al (1990) and Winter and Milstein (1991). Briefly, RPAS reagents were used to produce a cDNA library from mRNA through reverse transcription with random ($N_6$) primers. Separate antibody $V_H$- and $V_L$-chain-encoding regions were amplified by PCR from the cDNA library using $V_H$- and $V_L$-specific oligonucleotide primers. Isolated, agarose gel-purified $V_H$- and $V_L$-encoding DNA were spliced together by PCR using primers designed to introduce a linking sequence between the two gene segments and specific restriction sites at both 5′ (*SfiI*) and 3′ (*NotI*) ends of the spliced sequence. Restriction digestion with *SfiI* and *NotI* endonucleases and agarose gel purification of the digested linked product preceded ligation of this DNA into the *SfiI*- and *NotI*-digested phagemid vector pCANTAB 5E. This vector was then used to transform competent TG1 *E. coli* cells using a heat shock (42°C) transformation method (RPAS). Transformed TG1 *E. coli* were then subjected to a phage rescue protocol using the helper phage M13KO7, producing a recombinant phage library ($3 \times 10^9$ pfu) of C595 encoding $V_H$- and $V_L$-linked genes. This phage library was used to infect HB2151 *E. coli* to obtain clones able to produce soluble scFv as opposed to displaying the scFv as a fusion product on the phage surface. A colony of HB2151 cells was transferred from a minimal medium plate and grown for 3 h at 37°C, with shaking at 250 r.p.m. (RPAS). The phage library solution (1 µl – approximately $1 \times 10^6$ pfu) was added to the cells incubated at 37°C with intermittent shaking and the cells were streaked onto SOBAG-N plates (SOBAG medium containing 100 µg $ml^{-1}$ nalidixic acid; RPAS) and incubated overnight at 30°C. Individual colonies were then analysed by PCR to check for the presence of C595 $V_H$- and $V_L$-specific DNA inserts.

### Primer design

The primers used in the scFv assembly were provided in the RPAS mouse scFv module. Primers used in sequencing reactions and in PCR analyses were prepared using an ABI 394 DNA synthesizer (ABI, Foster City, CA, USA) at the Biopolymer Synthesis and Analysis Unit (BSAU), Department of Biochemistry, University of Nottingham, UK. The primers denoted S1 (5′-CAACGT-GAAAAAATTATTATTCGC-3′) and S6 (5′-GTAAATGAATTTT-CTGTATGAGG-3′) were based on the pCANTAB 5E vector sequence. The primers denoted I1 (5′-TGTGCAAGAGATCGG-GATGGTTAC-3′) and I2 (5′-ACTACTCCACTGCTGGCAG-TAATA-3′) were based on the complementarity-determining region (CDR)-3-encoding DNA sequences of the C595 heavy and light chains respectively (Denton et al, 1995).

### PCR analysis

A non-suppressor strain of *E. coli* (HB2151) was infected with the recombinant phage library and infected clones were anlaysed by PCR for the incorporation of C595 scFv-encoding DNA using C595 heavy- and light-chain-specific primers I1 and I2 and the vector-specific primers S1 and S6. PCRs were set up using phage-infected HB2151 *E. coli* colonies as a source of C595 scFv-specific DNA. PCRs were performed in 100 µl aliquots using 50 pM of each primer per reaction on an OmniGene Thermal Cycler Controller (Hybaid, Teddington, UK) and consisted of 30 cycles of 94°C for 45 s, 56°C for 45 s and 72°C for 90 s, representing denaturation, annealing and elongation temperatures respectively.

### Agarose gel electrophoresis

Agarose gels (1%) were prepared by dissolving NuSieve agarose (Flowgen, Sittingbourne, UK) in TBE buffer (0.09 M Tris-borate, 0.002 M EDTA). Ethidium bromide (0.2 µg $ml^{-1}$) was incorporated into the gel before pouring. Samples were diluted with DNA loading buffer, and electrophoresis was performed. DNA was visualized using a Spectroline TM-312A Ultra Violet Transilluminator, and photographs were recorded using a Polaroid DS-34 Direct Screen Instant Camera.

### scFv expression

Induction of scFv expression was achieved using the *lacZ* promoter of the pCANTAB 5E vector by the addition of isopropyl beta-D-thiogalactopyranoside (IPTG) substrate into SB medium supplemented with 100 µg $ml^{-1}$ ampicillin (Sambrook et al, 1989). A total of 800 ml of substrate was added. Clone 31 was chosen for scFv production on this scale, as preliminary SDS-PAGE and Western blot analyses using 50 ml of substrate had identified this clone as the highest expressor of scFv.

### C595 scFv clone 31 purification

Recombinant C595 scFv clone 31 was recovered from the supernatant of the bacterial broth, which was centrifuged at 48 000 g for 1 h. The supernatant was decanted through Whatman no. 1 filter paper, sodium azide was added to 0.02% (w/v) and the supernatant was processed through a 0.2-µm, 500-ml bottle filter unit (Costar, High Wycombe, UK). The supernatant was then applied to a peptide epitope affinity column consisting of Sepharose 4B (Pharmacia) conjugated to the peptide APDTRPAPG (Price et al, 1991). This conjugate contains the epitope (RPAP) recognized by the antibody C595, thus only immunoreactive scFv will bind to this column. The column was washed by the addition of phosphate-buffered saline (PBS) containing 0.02% (w/v) sodium azide and then connected to a G25 Sephadex desalting column. Elution of the bound scFv was performed by the application of 3 M sodium thiocyanate. Purified scFv concentrations in the eluent were determined spectrophotometrically at 280 nm using the Beer–Lambert law.

### Analysis of scFv clones by sodium dodecyl sulphate (SDS) polyacrylamide gel electrophoresis (SDS-PAGE) and Western blotting

Affinity-purified C595 scFv clone 31 samples were diluted 1:1 with SDS loading buffer [bromophenol blue (0.05% w/v), sucrose (40% w/v), EDTA (0.1 M, pH 8.0) and SDS (0.5% w/v)] and pretreated by boiling for 5 min before loading. SDS-PAGE was performed using a PhastSystem Separation and Control Unit (Pharmacia) in conjunction with PhastGel precast gels (homogeneous acrylamide, 12.5% w/v). Silver staining was performed using the PhastGel silver staining kit on the PhastSystem Development Unit. Western blotting was achieved onto nitrocellulose membrane (Biorad, Hemel Hempstead, UK) using the PhastTransfer semi-dry transfer kit and transfer buffer (25 mM Tris, 190 mM glycine in

© Cancer Research Campaign 1997

1:4 methanol–water). After transfer, the membrane was incubated for 1 h in 0.1% casein (w/v) in PBS to block non-specific binding sites. After four washes with PBS, the membrane was incubated with a 1:1000 solution of anti-E-Tag/horseradish peroxidase (HRPO) conjugate (Pharmacia) in PBS for 1 h. After four washes with PBS, the blot was developed using 3-amino-9-ethylcarbazole in 50 mM sodium acetate buffer pH 5.5, with the addition of hydrogen peroxide at 5 µl ml$^{-1}$ (3-AEC substrate).

### DNA sequencing analysis

The clone containing C595-encoding DNA and expressing the scFv as identified by Western blot (clone 31) was further analysed through DNA sequencing. This clone was grown overnight and plasmid (phagemid) DNA extracted using a Hybaid Recovery Kit (Hybaid), essentially using protocols of plasmid minipreparation as outlined in Sambrook et al (1989). Sequencing reactions were performed on an ABI 373A DNA Sequencer (ABI) using the PRISM DyeDeoxy Terminator Cycle Sequencing Kit (ABI) and I1, I2, S1 and S6 primers.

### Immunoreactivity by enzyme-linked immunosorbant assay (ELISA)

Microtitre plates (96-well, flat bottomed, Falcon 3912; Becton Dickinson, Oxford, UK) were coated with affinity-purified urinary mucin (Price et al, 1990) or with branched chain polylysine conjugate containing multiple copies of the peptide CAPDTRPAPG (Hudecz and Price, 1992) (added at 50 µl per well at 10 µg ml$^{-1}$ and incubated for 37°C for 18 h). A negative control antigen was coated to the wells in the same manner. This consisted of a MUC2-related peptide (TPTGTQTPTT) conjugated to bovine serum albumin (BSA). The wells were washed four times with PBS (pH 7.3) containing 0.05% (w/v) Tween 20 (PBS/Tween), and the remaining non-specific sites were blocked by the addition of 1% BSA for 1 h. After four washes in PBS/Tween, semi-logarithmic serial dilutions of C595 scFv (75 µg ml$^{-1}$) were added at 50 µl per well and incubated for 1 h. The plates were again washed four times and anti-E-tag/HRPO (Pharmacia) at 1:1000 in PBS was added, 50 µl per well, and incubated for 1 h. The plates were washed four times in PBS/Tween. A solution of 0.033% (w/v) 2,2′-azino-bis (3-ethylbenzothiazoline-6-sulphonic acid) in 0.1 M citrate phosphate buffer pH 4.0, with 33% (v/v) hydrogen peroxide added at 0.3 µl ml$^{-1}$ (ABTS substrate) was added to the wells at 100 µl per well. Analysis of colour development was performed over a 10 min period using a Milenia Kinetic Analyser (Diagnostic Products Corporation, Llanberis, UK).

### Flow cytometry

Breast carcinoma T47D cells were grown in RPMI 1640 medium in 75-cm$^3$ tissue culture-treated flasks (Falcon) supplemented with 2 mM L-glutamine and 10% heat-inactivated fetal bovine serum. Adherent cells were grown to subconfluence and harvested after incubation with 0.05% (w/v) trypsin/0.02% (w/v) EDTA at 37°C. Cells ($1 \times 10^5$ cells per sample in duplicate) were transferred to a 1.5-ml Eppendorf tube and centrifuged at 10 000 *g* for 30 s. The supernatant was removed and the pellet resuspended in 1% BSA (w/v) in PBS and incubated on ice for 1 h. The cells were washed three times with PBS and then resuspended in 100 µl of antibody solutions and incubated on ice for 1 h. After three washes in PBS, secondary antibodies were applied to each sample. Samples with murine antibodies as the primary layer were treated with rabbit anti-mouse Ig/FITC-conjugated antibodies (Dako, High Wycombe, UK) (100 µl of a 1:500 dilution in PBS). Samples containing C595 scFv were incubated with an equal amount of a mouse anti-E-Tag antibody (Pharmacia) for 1 h on ice, washed four times in PBS and incubated in a solution of the rabbit anti-mouse Ig/FITC-conjugated antibody (Dako) as described above. Samples were then washed four times in PBS and fixed in 1 ml of 0.5% formaldehyde (v/v) in PBS and analysed by FACScan (Becton Dickinson). Relative fluorescence values were calculated according to Schmid et al (1988).

### Immunoreactivity with synthetic MUC1 peptide

C595, C595 scFv and an irrelevant IgG molecule, the anti-MUC2 monoclonal 996 (100 µl at 10 µg ml$^{-1}$), were added to 1:5 (v/v) suspensions of 4B-CL Sepharose beads (0.5 ml) both with and without peptide (CAPDTRPAPG) conjugated to them. Incubations of antibody and fragments with the Sepharose beads were performed as described for incubations with the cells by FACScan analysis. After the appropriate secondary antibody layers had been added and the beads washed in PBS, ABTS substrate solution was added to the beads as described in the ELISA procedure (100 µl per sample). The beads were centrifuged and the ABTS supernatant added to a 96-well microtitre plate for end point analysis on the Milenia Kinetic Analyser. Samples were analysed in triplicate.

### Immunohistochemistry

Sequential 3-µm sections were cut from archival paraffin blocks of breast and transitional cell bladder tumours, dewaxed in xylene and rehydrated in graded alcoholic solutions. Endogenous peroxidase activity was blocked with 1% hydrogen peroxide in methanol for 10 min. Non-specific binding was blocked with normal swine serum diluted 1:10 for 10 min. Immunostaining was performed using a semiautomated immunostainer (Shandon Sequenza). The primary antibody was applied for 1 h. To detect scFv binding, specimens were incubated for 1 h with mouse anti-E-Tag (1:50) in PBS. Optimal antibody concentrations were determined by titration. Detection of antibody binding was performed using biotinylated goat anti-mouse/rabbit immunoglobulins followed by streptavidin/biotinylated horseradish peroxidase complex (Dako, StreptABC complex/HRP duet, mouse/rabbit). Staining was performed by addition of 0.025% diaminobenzidine (Sigma) diluted in 0.1 M Tris buffer (pH 7.6), with hydrogen peroxide as the substrate. Tissue sections were counterstained with haematoxylin, then rehydrated in graded alcohol solutions, cleared in xylene and mounted in DPX mountant. Negative controls were performed by omitting the primary antibody. Immunostaining was performed without any form of antigen retrieval.

### Fluorescence quenching

Fluorescence quenching experiments were performed using both C595 and C595 scFv against MUC1 protein core-related peptides. C595 antibody was diluted in PBS to a concentration of 0.12 µM and C595 scFv diluted likewise to a concentration of 0.27 µM. Peptides were diluted to 60 µM. The antibody and fragment solutions (2.5 ml) were filtered through a 0.2-µm membrane filter unit (Sartorius, Göttingen, Germany) and 2 ml of these solutions

© Cancer Research Campaign 1997

**Figure 1** Agarose gel of PCR products obtained from C595 scFv phagemid library-infected HB2151 clones. Upper lanes contain PCR products from clones using S1 and S6 primers and lower lanes contain PCR products from clones using I1 and I2 primers. In both cases, lane 1 contains molecular weight standards of 2000, 1500, 1000, 750, 500, 300, 150 and 50 bp (Sigma). Lanes 2–7 contain DNA amplified from clones 3, 17, 22, 31, 69 and 72 respectively pipetted into a 3-ml quartz cuvette (1-cm pathlength). The solution was excited at 290 nm and emitted light monitored at 345 nm using a Luminescence Spectrometer LS-5 (Perkin Elmer). Aliquots (2.5 μl) of the peptide solutions were added to the antibody or fragment solutions until maximum fluorescence quenching was observed. Calculations were performed according to the method of Eisen (1964) and the results analysed by the Scatchard method using the program InPlot (GraphPad Software, San Diego, CA, USA).

### RESULTS

### C595 scFv assembly and expression

Insertion of C595 $V_H$- and $V_L$-specific DNA in the scFv assembly process was analysed by PCR amplification of the DNA from phagemid-infected HB2151 *E. coli* clones grown on ampicillin plates. These tests were performed using plasmid (phagemid)-specific primers S1 and S6 and C595-specific primers I1 and I2.

Analysis using the S1 and S6 primers in a number of clones confirmed the insertion of DNA in the *SfiI* and *NotI* cloning site. These clones (numbered 3, 17, 22, 31 and 69) produced PCR products of approximately 950 bp in length (Figure 1, upper panel). The size of these products is consistent with the assembly of linked $V_H$ and $V_L$ into the phagemid vector at the *SfiI* and *NotI* cloning site.

Infection of the ligated phagemid into HB2151 *E. coli* was also reported by the presence of C595-specific DNA in these clones using the I1 and I2 primers (Figure 1, lower panel). Here, clones containing the C595 scFv-encoding DNA produce PCR products of approximately 400 bp, which is consistent with the assembly of linked $V_H$ and $V_L$ into the phagemid vector in the correct orientation.

All clones generating PCR products of a size not consistent with the insertion of linked $V_H$- and $V_L$-encoding DNA (the example given in Figure 1 is clone 72) with S1 and S6 primers failed to produce PCR products from reactions containing I1 and I2 primers.

The expression of soluble recombinant scFv in those clones containing C595 scFv-encoding inserts was analysed by SDS-PAGE and Western blotting. The pCANTAB 5E phagemid vector uses a reporter sequence known as E-Tag to show the presence of scFv. The positioning of the cloning site and the presence of an Amber stop codon downstream of the E-Tag reporter sequence means that in the non-repressor (HB2151) system, soluble recombinant scFvs contain the E-Tag sequence (GAPVPYPDPLEPR) towards their C-termini. A murine antibody/HRPO conjugate recognizing the E-Tag peptide sequence allows the presence of expressed scFv to be visualized on Western blots. The use of this reporter plus analysis of the product size through SDS-PAGE

**Figure 2** Affinity-purified C595 scFv as analysed by silver-stained SDS-PAGE (lanes a and b) and Western blot (lanes e and f) with molecular weight marker values indicated on the extreme left. Lanes (c) and (d) are silver stained SDS-PAGE and Western blot analyses of unbound column waste respectively

© Cancer Research Campaign 1997

**Table 1** C595 scFv clone 31 sequence

| |
|---|
| M A Q V Q L Q E S G G G L V Q P G G S L K L S C A A S G F T |
| E – – – V – – – – – – – – – – – – – – – – – – – – – – – |
| F S **S Y G M S** W V R Q T P D K R L E L V A **T I N S N G G S T** |
| – – – – – – – – – – – – – – – – – – – – – – – – – – – – – – |
| **Y Y P D S V K G** R F T I S R D N A K N T L Y L Q M S S L K S |
| – – – – – – – – – – – – – – – – – – – – – – – – – – – – – – |
| E D T A M Y Y C A R **D R D G Y D E G F D Y** W G P G T T V T V |
| – – – – – – – – – – – – – – – – – – – – – – – – |
| S S G G G G S G G G G S G G G G S D I E L T Q S P S I M S A |
| ←– – – – – – – – – – – – – – – – – – – –→ Q – V – – – – – – A – – – – |
| S P G E K V T M T C **S A S S S V S Y M H** W Y Q Q K S G T S P |
| – – – – – – – – – – – – – – – – – – – – – – – – – – – – – – |
| K R W I Y **D T S K L A S** G V P A R F S G S G S G T S Y S L T |
| – – – – – – – – – – – – – – – – – – – – – – – – – – – – – – |
| I S S M E A E D A A T Y Y C **Q Q W S S N P P T** F G G R T K L |
| – – – – – – – – – – – – – – – – – – – – – – – – – – G – – – |
| E L K R A A A *G A P V P Y P D P L E P R* A A |
| – |

Bold residues indicate CDRs. Variations from the C595 parental sequence are presented underneath the scFv sequence. The linker sequence is indicated with arrows and the E-Tag sequence is given in italics.

**Figure 3** ELISA of affinity-purified C595 scFv vs purified MUC1 urinary mucin (-○-), MUC1 peptide conjugate AK-CG (-●-), MUC2 peptide conjugated to BSA (-□-) and casein (-■-)

reveals the presence of intact recombinant scFv. Preliminary studies were performed whereby whole-cell extracts, periplasmic extracts and supernatants from cultures of clones 3, 17, 22, 31 and 69 were tested by SDS-PAGE and Western blotting for the presence of scFv. Only the clone numbered 31 expressed soluble recombinant scFv in sufficient quantities to be revealed in the periplasm and the supernatant by Western blotting and immunostaining (data not shown).

This clone was further analysed by DNA sequencing techniques using the primers S1, S6, I1 and I2 to identify the exact DNA sequence of the inserted C595 scFv-encoding region. Table 1 presents the translated primary sequence of the C595 scFv clone 31 and compares the relevant portions of the assembled recombinant fragment with those of the parent antibody (Denton et al, 1995). The portions of the scFv representing the variable regions are homologous with parental antibody sequences except for five conservative changes. These differences reflect the degenerate nature of the primers used in the RPAS PCRs and the fact that primers with sequences that are not exactly complementary are

**Figure 4** Immunoreactivity of C595 (**A**) and C595 scFv (**B**) by Sepharose matrix ELISA. In **A**, signals are obtained through the incubation of samples with: (a) blank Sepharose with anti-mouse Ig/HRPO only; (b) blank Sepharose with C595 and anti-mouse Ig/HRPO; (c) peptide Sepharose with anti-mouse Ig/HRPO only; (d) peptide Sepharose with C595 and anti-mouse Ig/HRPO; (e) blank Sepharose with 996 and anti-mouse Ig/HRPO; and (f) peptide Sepharose with 996 and anti-mouse Ig/HRPO. In **B**, signals are obtained through the incubation of samples with: (a) blank Sepharose with anti-E-Tag and anti-mouse Ig/HRPO; (b) blank Sepharose with C595 scFv, anti-E-Tag and anti-mouse Ig/HRPO; (c) peptide Sepharose with anti-E-Tag and anti-mouse Ig/HRPO; and (d) peptide Sepharose with C595 scFv, anti-E-Tag and anti-mouse Ig/HRPO

© Cancer Research Campaign 1997

**Figure 5** Scatchard analyses of MUC1-related synthetic peptides in solution to C595 (■) and C595 scFv (□) obtained through fluorescence quenching. MUC1-related peptides consist of an immunodominant region related peptide (APDTRPAPG), the p(1–25) peptide (TAPPAHGVTSAPDTRPAPGSTAPPA) and larger peptides based on 3 (60 mer) and 5.25 (105 mer) repeats of the 20 amino acid repeat sequence for the MUC1 mucin protein core. The MUC2-related peptide (TPTGTQTPTT) is based on the tandem repeat sequence for the protein core of this mucin and contains the epitope for the 996 antibody (TGTQ)

**Figure 6** FACScan analysis of the binding of C595 and C595 scFv to T47D cells. w6/32 is an anti-HLA-ABC positive control antibody, whereas 996 is a MUC2-specific negative control antibody. Cells alone have no antibodies added to the cells, and FITC and E-Tag+FITC represent the addition of these antibodies as appropriate negative controls for C595 and C595 scFv respectively

**Figure 7** Serial breast tissue sections with carcinoma in situ towards the left-hand side and micrometastatic foci towards the right-hand side of the microphotographs, showing detection when stained with C595 (**A**) and C595 scFv (**B**)

able to anneal to the cDNA template and prime the PCR. No differences occur within the CDRs of the C595 scFv when compared with the C595 sequence.

### Purification of C595 scFv clone 31

The production of C595 scFv clone 31 was scaled up from 50 ml per sample to 800 ml per sample for purification of the antibody fragment by peptide epitope affinity chromatography. Fractions of column eluent were collected as described and the scFv in the eluent was quantified spectrophotometrically from UV absorbance at 280 nm. Appropriate fractions were further analysed by SDS-PAGE and Western blotting (Figure 2). Lanes (a) and (b) in Figure 2 report the purification of a protein identified as a single band of approximately 30 kDa by SDS-PAGE. After Western blot analysis of the same samples, lanes (e) and (f) show that this protein contains the E-Tag sequence and identify it as C595 scFv. The proteins that do not bind to the affinity column are identified in lane (c). None of these proteins cross-react with the reagents used in the Western blot analysis (lane d). Only a single band, representing unbound scFv, can be seen in the column waste eluent.

### ELISA tests

Figure 3 illustrates the binding of affinity-purified C595 scFv clone 31 to MUC1 antigen, as well as to a MUC1-related peptide (AK-CG) containing the RPAP epitope recognized by C595. It does not bind to the MUC2-related peptide (TPTGTQTPTT) or to wells blocked with casein. The reactivity of the scFv is consistent with the binding specificities of other anti-MUC1 antibodies, against both the native mucin and peptide derivatives of the MUC1 protein core. Failure to react with the MUC2 sequence peptide is consistent with epitope mapping data (Denton and Price, 1995) for C595 as the RPAP epitope is not present in the MUC2 peptide conjugate.

© Cancer Research Campaign 1997 

### Immunoreactivity

The immunoreactivity of both C595 antibody and C595 scFv was further tested using a simple assay incorporating the Sepharose affinity matrix used in the construction of the peptide affinity chromatography column. Both bound peptide (CAPDTRPAPG) and blank Sepharose (no bound peptide) were used in this assay. Figure 4A indicates that C595 is able to bind to the peptide-linked Sepharose but does not bind to blank Sepharose. Both the anti-mouse secondary layer and a non-specific IgG antibody (996) do not bind to either matrix. The same matrices were used to test the binding of C595 scFv (Figure 4B). Clearly, the scFv is able to bind to the peptide CAPDTRAPAPG.

### Fluorescence quenching

The results of fluorescence quenching experiments are given in Figure 5. Scatchard analysis of C595 and C595 scFv reveal that both whole and antibody fragments possess the capacity to bind to different MUC1-related peptide analogues in solution, while failing to react with the MUC2-related peptide. For those analogues containing only one RPAP epitope, i.e. APDTRPAPG and p(1–25), the binding affinities of C595 and C595 scFv are similar. However, in peptide analogues containing more than one epitope (60 mer and 105 mer), the whole antibody shows a much higher binding capacity than the C595 scFv.

### FACScan analysis of scFv binding to T47D cells

The binding of whole antibody and scFv to T47D breast carcinoma cells is illustrated in Figure 6. Mean fluorescence and associated standard deviations are calculated from normal distribution curves, and these values are related to the autofluorescence of unlabelled cells. These results indicate that C595, C595 scFv and w6/32 (a positive control for human HLA-ABC) are capable of binding to T47D cells. A non-specific IgG-negative control antibody (996) does not bind to these cells, presumably because of the lack of expression of MUC2 on the surface of the T47Ds. None of the secondary and tertiary antibodies used in the FACScan procedure (E-Tag and FITC) bound directly to the cells.

### Immunohistochemistry

Figure 7A and B shows the ability of both C595 and C595 scFv to preferentially bind to breast tumour tissue sections. The cytoplasmic and surface staining pattern is characteristic of the expression of MUC1 mucins as described by Zotter et al (1988). Some weak staining to benign breast ducts using the whole antibody is seen in Figure 7A but is not observed for the scFv (Figure 7B).

### DISCUSSION

The results presented here confirm the successful production of a recombinant single-chain antibody fragment (scFv) retaining the capacity to bind to MUC1 mucin. Assembly of scFv, using the antibody variable region genes and the recombinant antibody phage system as described, produces a recombinant protein in which the structural features that define the fine specificity of the parent antibody for its antigen are retained. The only observed differences in primary structure between the parent and recombinant variable regions occur at the priming sites for the antibody genes (Table 1). These reflect the presence of a degenerate primer mixture used in the RPAS to amplify genes from many V region gene families. The variation from the parental in the C595 scFv clone 31 is conservative and does not occur in the complementarity determining regions. These framework substitutions have no significant effect on antibody binding, nor does the incorporation of the $(G_4S)_3$ linker sequence and the reporter (E-Tag) sequence at the C-terminus. The ability to produce recombinant C595 scFv from this vector system should afford analyses of how point mutations in the CDRs affect antigen/antibody interactions, with particular emphasis on the CDR $H_3$. Use of these manipulations and the ability to rapidly analyse their effects on peptide antigen systems should give valuable information on how these antibodies recognize tumour cells, and these experiments are currently underway.

Analysis by FACScan and immunohistochemistry report the ability of the scFv to bind to tumour cells. Immunohistochemical analysis of the binding of scFv by these methods does not indicate binding of this molecule to benign breast ducts, an observation seen with the whole antibody. This may afford the preferential use of the scFv over the C595 whole antibody as a diagnostic agent. However, further immunohistochemical analyses are required to test this proposal and these investigations are being pursued. Similar staining patterns were obtained with sections from bladder carcinomas.

Fluorescence quenching (FQ) experiments indicate the capacity of the scFv to bind to MUC1 and different MUC1-related peptides. FQ analysis is sufficient to observe the general binding trends of these antibodies and fragments to peptides in solution. Techniques such as surface plasmon resonance may be used in future experiments to investigate binding constants of these antibodies and fragments on immobilized antigens and would serve to confirm the binding trends observed with FQ. From the experimental data presented, it is interesting to note the possible effect of avidity on the binding capacities of these reagents as observed in the multiple epitopic peptide antigens (60 and 105 mer). This may indicate that a recombinant bivalent scFv (diabody) may prove to be a useful reagent in a diagnostic scenario, and the production of such a molecule is currently being pursued. However, a low-affinity reagent may also be of some use as affinity influences the penetration of these reagents into solid tumours. This property allied to the reduced size of the scFv and the penetrative advantage that this gives (Yokota et al, 1992) may be of benefit in the treatment of more substantial tumours.

Experiments have been performed in which intravesicular administration of $^{111}$In-labelled C595 in patients with superficial bladder cancer resulted in the selective accumulation of the antibody in the tumour with a mean tumour–normal selectivity ratio of 12:1 after 2 h. Administration of whole mouse antibody by this route has been shown not to produce human anti-murine antibody (HAMA) responses in patients even after multiple administrations (Kunkler et al, 1995). These findings establish the basis for extending the clinical usage of anti-MUC1 monoclonal antibodies as targeting agents with anti-tumour activity. A clinical trial is presently underway to examine tumour targeting of $^{67}$Cu-labelled C595 following intravesical administration in patients with superficial bladder cancer (Hughes et al, 1997). This radionuclide emits both gamma and beta radioactivity and thus has potential use for both diagnosis and radioimmunotherapy. The increased tumour penetrative properties that C595 scFv may offer is presently being examined.

Current trials are also underway whereby whole [$^{111}$In] C595 is administered systemically. An obvious advantage of using the

© Cancer Research Campaign 1997

scFv to replace the whole antibody would be the decrease in immunogenicity of the fragment over the parent molecule as well as the pharmacokinetic advantage of using a smaller molecule that has increased blood clearance times. Although still of murine origin, a decrease in immunogenicity may allow multiple therapeutic doses to be administered, an obvious advantages in patient management.

In summary, recombinant C595 scFv retains the ability to bind to tumour cells, MUC1 antigen and synthetic MUC1 peptides. This reagent, therefore, has diagnostic and therapeutic potential. It may further prove to be a valuable tool in probing the fine specificity of antibody recognition on MUC1-positive tumour cells, leading to a greater understanding of the immune recognition of cancer cells.

### ACKNOWLEDGEMENTS

We thank John Ronan (Department of Histopathology, City Hospital, Nottingham) for immunohistochemical analyses and Dr Adrian Robins (Department of Immunology, University of Nottingham) for FACScan analyses. Dr J Darrell Fontenot (Los Alamos National Laboratory, NM, USA) is thanked for providing the 60 mer and 105 mer peptides. These studies were supported by a Project Grant (No. 2168) awarded by the Cancer Research Campaign.

### REFERENCES

Berruti A, Tampellini M, Torta M, Buniva T, Gorzegno G and Dogliotti L (1994) Prognostic value in predicting overall survival of two mucinous markers: CA 15-3 and CA 125 in breast cancer patients at first relapse of disease. *Eur J Cancer* **30A**: 2082–2084

Denton G and Price MR (1995) Immune responses to the MUC1 mucin. *Pathol Oncol Res* **1**: 27–31

Denton G, Davies GM, Scanlon MJ, Tendler SJB and Price MR (1995) Primary sequence determination and molecular modelling of the variable region of an anti-MUC1 mucin monoclonal antibody. *Eur J Cancer* **31A**: 214–221

Dixon AR, Price MR, Hand CW, Sibley PEC, Selby C and Blamey RW (1993) Epithelial mucin core antigen (EMCA) in assessing therapeutic response in advanced breast cancer – a comparison with CA15.3. *Br J Cancer* **68**: 947–949

Domenech N, Henderson RA and Finn OJ (1995) Identification of an HLA-A11 restricted epitope from the tandem repeat domain of the epithelial tumour-antigen mucin. *J Immunol* **155**: 4766–4774

Eisen HN (1964) Determination of antibody affinity for haptens and antigens by means of fluorescence quenching. In *Methods in Medical Research*, Vol X, Eisen HN. (ed.), pp. 115. Year Book Medical Publishers: Chicago

Gendler S, Taylor-Papadimitriou J, Duhig T, Rothbard J and Burchell J (1988) A highly immunogenic region of a human polymorphic epithelial mucin expressed by carcinomas is made up of tandem repeats. *J Biol Chem* **263**: 12820–12823

Gendler SJ, Spicer AP, Lalani AL, Duhig T, Peat N, Burchell J and Pemberton M (1991) Structure and biology of a carcinoma-associated mucin, MUC1. *Am Rev Resp Dis* **144**: S42–47

Hudecz F and Price MR (1992) Monoclonal antibody binding to peptide epitopes conjugated to synthetic branched chain polypeptide carriers. *J Immunol Methods* **147**: 201–210

Hughes ODM, Bishop MC, Perkins AC, Frier M, Price MR, Denton G, Smith A, Rutherford R andSchubiger PA (1997) Preclinical evaluation of copper-67 labelled anti-MUC1 mucin for therapeutic use in bladder cancer. *Eur J Nucl Med* **24**: 439–443

Jerome KR, Barnd DL, Bendt KM, Boyer CM, Taylor-Papadimitriou J, McKenzie IFC, Bast RC and Finn OJ (1991) Cytotoxic T-lymphocytes derived from patients with breast adenocarcinoma recognise an epitope present on the protein core of a mucin molecule preferentially expressed by malignant cells. *Cancer Res* **51**: 2908–2916

Köhler G and Milstein C (1975) Continuous cultures of fused cells secreting antibody of predefined specificity. *Nature* **256**: 495–497

Kotera Y, Fontenot D, Pecher G, Metzgar RS and Finn OJ (1994) Humoral immunity against a tandem repeat epitope of human mucin MUC-1 in sera from breast, pancreatic and colon cancer patients. *Cancer Res* **54**: 2856–2860

Kunkler RB, Bishop MC, Green DJ, Pimm MV, Price MR and Frier M (1995) Targeting of bladder cancer with radiolabelled monoclonal antibody NCRC-48 – a novel approach for intravesical therapy. *Br J Urol* **75**: 9–17

Martoni A, Zamagni C, Bellanova B, Zanichelli L, Vecchi F, Cacciari N, Strocchi E and Pannuti F (1995) CEA, MCA CA 15.3 and CA 549 and their combinations in expressing and monitoring metastatic breast cancer: a prospective comparative study. *Eur J Cancer* **31A**: 1615–1621

McCafferty J, Griffiths AD, Winter G and Chiswell DJ (1990) Phage antibodies – filamentous phage displaying antibody variable domains. *Nature* **348**: 552–554

Perkins AC, Symonds IM, Pimm MV, Price MR, Wastie ML and Symonds EM (1993) Immunoscintigraphy of ovarian carcinoma using a monoclonal antibody (In-111-NCRC-48) defining polymorphic epithelial mucin. *Nucl Med Commun* **14**: 578–586

Price MR and Tendler SJB (1993) Polymorphic epithelial mucins (PEM): molecular characteristics and association with breast cancer. *Breast* **2**: 3–7

Price MR, Pugh JA, Hudecz F, Griffiths W, Jacobs E, Clarke AJ, Chan WC and Baldwin RW (1990) C595 – a monoclonal antibody against the protein core of human urinary epithelial mucin commonly expressed in breast carcinomas. *Br J Cancer* **61**: 681–686

Price MR, Sekowski M and Tendler SJB (1991) Purification of anti-epithelical mucin monoclonal antibodies by epitope affinity chromatography. *J Immunol Meth* **139**: 83–90

Price MR, Briggs S, Scanlon MJ, Tendler SJB, Sibley PEC and Hand CW (1992) The mucin antigens – what are we measuring? *Disease Markers* **9**: 205–212

Rughetti A, Turchi V, Ghetti CA, Scambia G, Panici PB, Roncucci G, Mancuso SM, Frati L and Nuti M (1993) Human B-cell immune response to the polymorphic epithelial mucin. *Cancer Res* **53**: 2457–2459

Sambrook J, Fritsch EF and Maniatis T (1989) *Molecular Cloning: a Laboratory Manual*, 2nd edn. Cold Spring Harbor Press: Cold Spring Harbor, NY

Schmid I, Schmid P and Giorgi JV (1988) Conversion of logarithmic channel numbers into relative linear fluorescence intensity. *Cytometry* **9**: 533–538

Symonds IM, Price MR, Pimm MV, Perkins AC, Wastie ML, Baldwin RW and Symonds EM (1992) Preliminary report of tumour localisation and imaging of ovarian neoplasia with a new monoclonal antibody raised against urinary mucin. In *Tumour Associated Antigens. Oncogenes, Receptors, Cytokines, in Tumour Diagnosis and Therapy at the Beginning of the Nineties*, Klapdor R. (ed.) pp. 572–577. W Zuckschwerdt: Munich

Winter G and Milstein C (1991) Man made antibodies. *Nature* **349**: 293–299

Yokota T, Milenic DE, Whitlow M and Schlom J (1992) Rapid tumor penetration of a single chain Fv and comparison with other immunoglobulin forms. *Cancer Res* **52**: 3402–3408

Zotter S, Hageman PC, Lossnitzer A, Mooi WJ and Hilgers J (1988) Tissue and tumour distribution of human polymorphic epithelial mucin. *Cancer Rev* **11–12**: 55–79

© Cancer Research Campaign 1997